# Painful Paroxysmal Dystonia as a Revealing Symptom of Multiple Sclerosis: A Case Report and Literature Review

**DOI:** 10.7759/cureus.98568

**Published:** 2025-12-06

**Authors:** Fatima Ez-Zahra Mabrouki, Samah Yousfi, Sanae Elhasnaoui, Yassine Mebrouk

**Affiliations:** 1 Department of Ophthalmology, Faculty of Medicine and Pharmacy, Mohammed VI University Hospital, Oujda, MAR; 2 Department of Neurology, Faculty of Medicine and Pharmacy, Mohammed VI University Hospital, Oujda, MAR

**Keywords:** corticosteroid therapy, ephaptic transmission, inaugural symptom, mri lesions, multiple sclerosis, painful movement disorders, paroxysmal dystonia

## Abstract

Multiple sclerosis (MS) can present with a wide range of motor symptoms, including movement disorders that are often underrecognized. Among these, painful paroxysmal dystonias, though rare, are particularly important to recognize when they occur as an initial manifestation. We report the case of a 51-year-old woman with no significant past medical history who presented with recurrent, brief, painful dystonic movements affecting the right upper limb and hemiface. Magnetic resonance imaging (MRI) revealed demyelinating lesions consistent with MS, and clinical improvement was achieved with corticosteroid therapy. Early recognition of such presentations is essential for accurate diagnosis and timely management.

## Introduction

Multiple sclerosis (MS) is a chronic inflammatory and demyelinating disease of the central nervous system, marked by neuroinflammatory and neurodegenerative processes [1]. While its classic presentation involves motor or sensory deficits, visual loss, or cerebellar signs, a broader spectrum of paroxysmal phenomena has been documented, including painful dystonic episodes [2,3].

Painful paroxysmal dystonia, though underrecognized, may constitute an inaugural sign of MS and is frequently mistaken for focal seizures, leading to inappropriate workup and delays in diagnosis [4-6]. This report highlights the diagnostic value of such dystonias through a representative case, supplemented by a literature review on their pathophysiological mechanisms, imaging correlations, and treatment approaches.

## Case presentation

**Figure 1:**
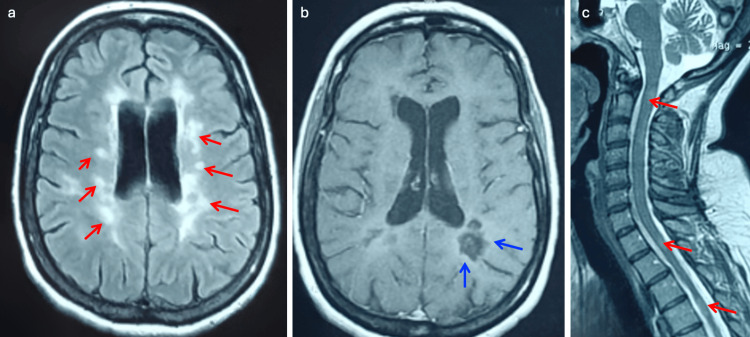
Brain (axial T2-FLAIR (a) and post-contrast T1 (b)) and cervical spinal cord (sagittal T1 (c)) MRI revealing multiple demyelinating lesions, including an enhancing lesion in the right centrum semiovale FLAIR: fluid-attenuated inversion recovery

A previously healthy, 51-year-old woman presented with brief, stereotyped, and intensely painful dystonic spasms affecting the right upper limb and hemiface. These episodes lasted only a few seconds but occurred frequently. The patient also reported a history of transient neurological symptoms with spontaneous resolution, suggestive of prior demyelinating events. These included episodes of visual blurring and paresthesias involving the right hemibody. Neurological examination revealed quadripyramidal signs, including spasticity, hyperreflexia, and posterior cord syndrome. Neuroimaging was performed to investigate the possibility of a basal ganglia lesion that could explain the clinical presentation. Magnetic resonance imaging (MRI) of the brain and spinal cord revealed multiple supratentorial and cervical demyelinating lesions, including enhancing lesions in the right centrum semiovale (Figure 1).

Oligoclonal bands were detected in the cerebrospinal fluid (CSF). Taken together, the clinical presentation, MRI findings of the brain and spinal cord, and the presence of oligoclonal bands in the CSF were consistent with a diagnosis of MS. A five-day course of intravenous methylprednisolone resulted in complete clinical remission, with the disappearance of the dystonic episodes. A disease-modifying therapy with fingolimod was subsequently initiated. After 15 months of treatment, the patient has experienced no clinical relapses, and her Expanded Disability Status Scale (EDSS) score remains at 1.

## Discussion

Paroxysmal dystonia in MS is a rare phenomenon, with prevalence rates reported below 2% [7-9]. These episodes are characterized by sudden, involuntary, often painful muscle contractions that result in transient abnormal postures or movements. The episodes are typically brief, lasting from seconds to minutes, and may recur frequently, up to 100 times per day [7,9,10].

Historically termed “painful tonic spasms,” these movements are triggered by voluntary actions, sensory stimuli, or even hyperventilation [7,9,11]. They often lead to diagnostic confusion with focal seizures or other paroxysmal movement disorders.

The pathophysiology is believed to involve ephaptic transmission in demyelinated axons, particularly within the corticospinal tract [7,9-11]. Osterman et al. proposed that demyelinated fibers may abnormally discharge due to disrupted myelin integrity, allowing adjacent axons to cross-excite [9,11]. Inflammatory activity may exacerbate this process, explaining the rapid response to corticosteroids in many cases, including ours.

Imaging studies have identified possible involvement of the internal capsule, thalamus, basal ganglia, brainstem, and cervical spinal cord in generating these dystonic spasms [8,10,11]. However, no consistent radiological-clinical correlation has been universally validated, emphasizing the complex and multifocal nature of MS-related movement disorders.

The differential diagnosis includes epilepsy, tetany, stiff-person syndrome, and psychogenic movement disorders. In our patient, the episodic nature, stereotypy, absence of electroencephalogram (EEG) abnormalities, and response to steroids favored an MS-related paroxysmal dystonia rather than epileptic or functional origin.

Therapeutic options extend beyond corticosteroids. Antiepileptic drugs, such as carbamazepine, oxcarbazepine, levetiracetam, and phenytoin, have shown benefit in various series [9-11]. Acetazolamide, clonazepam, botulinum toxin, and even cannabis-based therapies have also been used with variable success [7,9-11]. In some cases, symptoms resolve spontaneously within weeks.

Ultimately, identification of dystonic paroxysms as an MS manifestation is critical. Their appearance may signal active demyelination and justify not only symptomatic management but also initiation or escalation of disease-modifying therapies [12].

## Conclusions

Painful paroxysmal dystonias, though rare, are a clinically significant manifestation of MS. When appearing as inaugural symptoms, they pose diagnostic challenges and are often misinterpreted. Early recognition is essential to prevent diagnostic delays and to ensure the timely initiation of appropriate therapy. Corticosteroids and antiepileptics are effective in most cases.
